# Challenges in estimating the counterfactual placebo HIV incidence rate from a registration cohort: The PrEPVacc trial

**DOI:** 10.1177/17407745241304721

**Published:** 2024-12-31

**Authors:** Sheila Kansiime, Christian Holm Hansen, Eugene Ruzagira, Sheena McCormack, Richard Hayes, David Dunn

**Affiliations:** 1 Medical Research Council/Uganda Virus Research Institute and The London School of Hygiene & Tropical Medicine, Uganda Research Unit, Entebbe, Uganda; 2 Medical Research Council International Statistics and Epidemiology Group, The London School of Hygiene & Tropical Medicine, London, UK; 3 Medical Research Council Clinical Trials Unit, University College London, London, UK

**Keywords:** HIV, pre-exposure prophylaxis, sub-Saharan Africa, active-controlled trials, counterfactual placebo HIV incidence, novel HIV trial designs

## Abstract

**Background:**

There is increasing recognition that the interpretation of active-controlled HIV prevention trials should consider the counterfactual placebo HIV incidence rate, that is, the rate that would have been observed if the trial had included a placebo control arm. The PrEPVacc HIV vaccine and pre-exposure prophylaxis trial (NCT04066881) incorporated a pre-trial registration cohort partly for this purpose. In this article, we describe our attempts to model the counterfactual placebo HIV incidence rate from the registration cohort.

**Methods:**

PrEPVacc was conducted at four study sites in three African countries. During the set up of the trial, potential participants were invited to join a registration cohort, which included HIV testing every 3 months. The trial included a non-inferiority comparison of two daily, oral pre-exposure prophylaxis regimens (emtricitabine/tenofovir disoproxil fumarate, emtricitabine/tenofovir alafenamide fumarate), administered for a target duration of 26 weeks (until 2 weeks after the third of four vaccinations). We developed a multi-variable Poisson regression model to estimate associations in the registration cohort between HIV incidence and baseline predictors (socio-demographic and behavioural variables) and time-dependent predictors (calendar time, time in follow-up). We then used the estimated regression coefficients together with participant characteristics in the active-controlled pre-exposure prophylaxis trial to predict a counterfactual placebo incidence rate. Sensitivity analyses regarding the effect of calendar period were conducted.

**Results:**

A total of 3255 participants were followed up in the registration cohort between July 2018 and October 2022, and 1512 participants were enrolled in the trial between December 2020 and March 2023. In the registration cohort, 106 participants were diagnosed with HIV over 3638 person-years of follow-up (incidence rate = 2.9/100 person-years of follow-up (95% confidence interval: 2.4–3.5)). The final statistical model included terms for study site, gender, age, occupation, sex after using recreational drugs, time in follow-up, and calendar period. The estimated effect of calendar period was very strong, an overall 37% (95% confidence interval: 19–51) decline per year in adjusted analyses, with evidence that this effect varied by study site. In sensitivity analyses investigating different assumptions about the precise effect of calendar period, the predicted counterfactual placebo incidence ranged between 1.2 and 3.7/100 person-years of follow-up.

**Conclusion:**

In principle, the use of a registration cohort is one of the most straightforward and reliable methods for estimating the counterfactual placebo HIV incidence. However, the predictions in PrEPVacc are complicated by an implausibly large calendar time effect, with uncertainty as to whether this can be validly extrapolated over the period of trial follow-up. Other limitations are discussed, along with suggestions for mitigating these in future studies.

## Introduction

Pre-exposure prophylaxis (PrEP) is a drug regimen taken by HIV-negative persons to prevent the acquisition of HIV. Oral emtricitabine/tenofovir disoproxil fumarate (Truvada) has been shown to be highly effective, reducing the risk of infection by around 90%–95% if taken as indicated.^1,2^ This has complicated the evaluation of alternative experimental PrEP agents, since the very high efficacy of Truvada effectively rules out the use of placebo-controlled trials.^3^ Instead, experimental agents have had to be assessed using an active-controlled, non-inferiority design, with Truvada as the comparator arm.^4^ However, these trials require very large sample sizes to yield sufficient incident HIV infections (the primary endpoint) to make reliable inferences. For example, more than 15,000 person-years of follow-up (PYFU) are needed when assuming a background incidence rate of 4/100 PYFU and aiming to show ≥50% preservation of effect in comparison to a standard intervention which provides 80% protection.^4^ Trials of such magnitude are generally unfeasible, partly because they are very costly, and because it is increasingly difficult to identify populations at high risk of HIV infection. This can result in the recruitment of populations at lower risk, ultimately leading to inconclusive results due to insufficient statistical power.

This problem has stimulated major methodological developments in HIV prevention research. Central to this work has been an appreciation of the critical importance of estimating the ‘counterfactual placebo incidence’, that is, the HIV incidence that would have been observed if the trial had included a placebo control arm. This has also been referred to as the hypothetical background HIV incidence rate in the trial population. Various options have been suggested for how a counterfactual placebo HIV incidence rate might be estimated.^4–8^ Some of the commonly suggested approaches include statistical modelling informed by previous or concurrent studies,^9^ using recency assay estimates,^6^ using the adherence-efficacy relationship of Truvada,^10^ and using sexually transmitted infections data (based on the known correlation with HIV incidence).^11^ All the suggested approaches have considerable challenges with no clearly preferred approach.^12^ To date, very few clinical trials have used these approaches and so experience in their use is limited.^8^

In this article, we describe our experience with using data from a pre-trial registration cohort. This cohort was established during the setup phase of the PrEPVacc trial (NCT04066881) to facilitate participant enrolment in the trial and to determine the target sample size at each trial site. The cohort also provided an opportunity to estimate the counterfactual placebo HIV incidence. The PrEPVacc trial used a factorial design to compare two different PrEP regimens: emtricitabine/tenofovir disoproxil fumarate (Truvada) and emtricitabine/tenofovir alafenamide fumarate (Descovy) and to evaluate two alternative HIV vaccine regimens versus placebo. The analyses presented in this article are informative for the comparison of the two PrEP regimens. Although we anticipated that extrapolation of the HIV incidence rate from the registration cohort to the trial population would be relatively straightforward, we observed strong calendar time effects which necessitated the fitting of a range of statistical models.

## Materials

### Study design and population

PrEPVacc was conducted at four study sites in three countries: Masaka, Uganda; Mbeya and Dar es Salaam, Tanzania; and Durban, South Africa. Individuals were eligible to participate in the registration cohort if they were HIV-negative adults (18–45 years), at high-risk of HIV infection and consented to the study procedures. Enrolled individuals were followed for up to 3 years with clinic visits every 3 months, where they received HIV testing and counselling and completed interviewer-administered questionnaires on behavioural and other HIV risk factors. PrEP uptake during follow-up was monitored. PrEP was available on-site in Durban, and through referral to a local provider in Masaka from 2018. PrEP became available through referral to local providers in Mbeya and Dar es Salaam in early to mid 2021.

**Figure 1.**
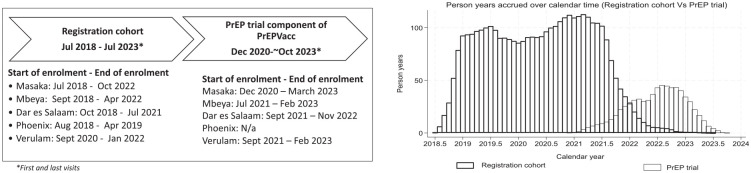
Registration cohort and trial, enrolment dates and person years accrued over time.

Enrolment in the registration cohort took place between July 2018 and October 2022, depending on study site (Figure 1). Enrolment in the PrEPVacc trial and randomisation to study arms took place between December 2020 and March 2023. Until February 2022, individuals needed first to be enrolled in the registration cohort prior to recruitment into the trial; however, this restriction was lifted to allow direct recruitment from the study communities into the trial. Follow-up of cohort participants generally continued for 3 months or less once enrolments into the trial started at a site. Enrolment in the registration cohort was also conducted at a site in Maputo, Mozambique. However, this site did not participate in the trial^13^ and has been excluded from the current analysis. In Durban, South Africa, recruitment to the registration cohort was initially conducted at the Phoenix site, and later the Verulam site (they are approximately 10–15 km apart). Recruitment for the trial did not occur in Phoenix; however, the site’s registration cohort data were included in the current analyses because of the close similarity of its participants with those at the Verulam site.

In the trial, participants were randomised 1:1:1 to receive one of two HIV vaccine regimens or saline placebo at weeks 0, 4, 24, and 48.^14,15^ Participants were also concurrently randomised to one of two oral PrEP regimens: once daily Truvada or Descovy, for a target duration of 26 weeks (until 2 weeks after the third vaccination). The rationale for a limited duration of PrEP was partly because any clinical protective effect of the vaccine was not anticipated until after the third vaccination. After the PrEP component of the trial, willing participants were able to access PrEP locally. Participants were followed for a minimum of 74 weeks. The primary endpoint for the analysis of both the vaccine and PrEP interventions is confirmed incident HIV infection. HIV infections diagnosed at or before 2 weeks after the third vaccination contribute to the primary PrEP analysis, while infections occurring thereafter contribute to the primary vaccine analyses. In this article, we focus exclusively on the follow-up relevant to the PrEP analysis.

The primary estimand for the PrEP analysis is the averted infections ratio (AIR) comparing Descovy (experimental regimen) versus Truvada (control regimen). Rather than comparing the two groups in terms of observed events, the AIR compares the number of events averted by the two treatments.

This measure has a natural preservation-of-effect interpretation and allows more powerful inference on non-inferiority than the traditional metrics, the rate ratio or the rate difference.^16^ However, calculation of the AIR requires an estimate of the counterfactual placebo HIV incidence rate. (Of note, this article does not include estimations of the AIR, which will require HIV incidence data observed in the PrEPVacc trial. Analysis of the AIR will be presented in a subsequent paper).

Finally, we note that the registration cohort and trial overlapped with the COVID-19 epidemic. The trial continued as planned, but HIV transmission is likely to have been impacted by lockdown measures. These differed both by degree and calendar period in the three countries, although for simplicity, we have defined the COVID-19-affected period as extending from March 2020 to July 2021.

### Statistical analysis

Analyses were conducted in Stata version 17.0 (College Station, TX, US), using data as of 31 January 2024. Analyses for associations between HIV incidence and key socio-demographic variables, behavioural risk indicators and time-related predictors within the registration cohort have been conducted and published previously.^17^ In this article, these analyses have been extended to make extrapolated predictions about the counterfactual placebo HIV incidence in the trial.

Estimating the counterfactual placebo HIV incidence rate was a two-stage process: (a) univariable and multi-variable Poisson regression models were fitted, and forward stepwise model building was used to derive the final multi-variable model, with both baseline predictors (socio-demographic and behavioural variables) and time-dependent predictors (calendar period, time in follow-up) added to the model if they had a P value < 0.2. Gender, age and site were added a priori. Lexis expansions were used to create categories for time in follow-up and calendar period. (b) Once a suitable predictive model was identified, trial participants’ baseline data and their entry and censoring dates were applied to the fitted model to predict a counterfactual placebo HIV incidence rate. Ninety-five percent confidence intervals (CIs) were estimated using bootstrapping, resampling participants with replacement from the registration cohort and repeatedly re-estimating the parameters of the previously identified prediction model. A total of 1000 bootstrap samples were run for each estimate, and the 2.5th and 97.5th percentiles from the empirical distribution were reported.

Sensitivity analyses were conducted making alternative assumptions about the effect of calendar period on HIV incidence. These included: (1) assuming a null effect of calendar period; (2) assuming site-specific calendar period effects; (3) assuming region-specific (East Africa vs South Africa) calendar period effects; (4) assuming a uniform calendar period effect across sites (ie no site calendar period interaction term); (5) adjusting for a binary COVID-19 effect, and (6) excluding registration cohort data from 2018 to 19 as this is less proximal to the period of trial follow-up. An additional sensitivity analysis excluded participants who ever used PrEP in the registration cohort.

## Results

### Characteristics and follow-up of cohort and trial participants

A total of 3255 participants were enrolled in the registration cohort between July 2018 and October 2022 and had at least one follow-up HIV test. As of the 31 January 2024 data extract, 1512 participants had been enrolled in the trial between December 2020 and March 2023, and of these, 1386 had been dispensed PrEP and had at least one follow-up HIV test in the PrEP component of the trial.

Figure 1 shows the overlap in follow-up, by calendar time, between the registration cohort and the trial. Enrolment and follow-up in the cohort declined once the sites opened for enrolment in the trial. This left a relatively brief period of overlap in follow-up between the cohort and the trial, extending from around early-2021 to mid-2022.

At enrolment (Table 1), 84% of registration cohort participants were female, 46% were older than 24 years, 60% reported being sex workers, 11% reported being salon/lodge/bar workers, and 62% were single. Around 58% had 1 year or less follow-up in the cohort, 20% between 1 and 2 years, and 22% more than 2 years. Among participants enrolled in the trial, 598 (43%) were recruited directly without any follow-up in the registration cohort. Of the remaining 788, most had either less than 6 months follow-up (312, 40%) or 6–12 months follow-up (267, 34%) in the cohort prior to randomisation. Demographically, trial participants were broadly similar to cohort participants (Table 1): 87% were female, 53% were older than 24 years, 60% reported being sex workers, 5% reported being salon/lodge/bar workers, and 41% were single. Only 13% of trial participants were enrolled in Dar es Salaam, a smaller proportion compared to the registration cohort. Apart from a higher proportion of participants in the trial than in the cohort reporting a diagnosis of or treatment for a sexually transmitted infection in the last 3 months at baseline (26% vs 18%), there were no notable differences in reported behavioural risk indicators.

**Table 1.** A comparison of demographic and risk indicator characteristics between participants in the registration cohort and the PrEPVacc trial.

Characteristic	PrEPVacc registration cohort^a^				PrEPVacc trial	
	N (%)	HIV infections	PY	IR/100 PY (95% CI)	N (%)	PY
Overall	3255 (100)	106	3638.1	2.9 (2.4–3.5)	1386	703.6
**Time between cohort enrolment and trial enrolment (months)**						
0 (N/A)^b^	N/a	N/a	N/a	N/a	598 (43)	284.8
0.1–6.0	N/a	N/a	N/a	N/a	312 (23)	160.1
6.1–12.0	N/a	N/a	N/a	N/a	267 (19)	149.8
12.1–18.0	N/a	N/a	N/a	N/a	100 (7)	52.4
18.1–36.0	N/a	N/a	N/a	N/a	109 (8)	56.5
**Time in follow-up (years) in the PrEPVacc study** ^c^						
0.00–1.00 years	3255 (100)	65	2227.4	2.9 (2.3–3.7)	1177 (85)	521.0
1.01–2.00 years	1362 (42)	36	983.5	3.7 (2.6–5.1)	409 (30)	132.8
2.01 or higher	702 (22)	5	427.2	1.2 (0.5–2.8)	113 (8)	49.8
**Calendar period** ^c^						
2018	1119 (34)	6	184.0	3.3 (1.5–7.3)	0 (0)	0.0
2019	2227 (68)	49	1132.9	4.3 (3.3–5.7)	0 (0)	0.0
2020	2529 (78)	33	1165.5	2.8 (2.0–4.0)	4 (0)	0.2
2021	3101 (95)	15	1167.2	1.3 (0.8–2.1)	415 (30)	123.9
2022/2023	2382 (73)	3	692.2	0.4 (0.1–1.3)	1287 (93)	579.7
**Site**						
Dar es Salaam, Tanzania	904 (28)	35	1606.3	2.2 (1.6–3.0)	179 (13)	92.6
Masaka, Uganda	1122 (34)	24	897.8	2.7 (1.8–4.0)	414 (30)	211.1
Phoenix/Verulam, South Africa^d^	546 (17)	12	372.0	3.2 (1.8–5.7)	357 (26)	187.2
Mbeya, Tanzania	683 (21)	35	762.1	4.6 (3.3–6.4)	436 (31)	212.7
**Gender**						
Male	519 (16)	6	580.1	1.0 (0.5–2.3)	180 (13)	93.0
Female	2736 (84)	100	3058.0	3.3 (2.7–4.0)	1206 (87)	610.6
**Age**						
≤24 years	1771 (54)	66	1904.5	3.5 (2.7–4.4)	649 (47)	321.8
>24 years	1484 (46)	40	1733.6	2.3 (1.7–3.1)	737 (53)	381.8
**Occupation**						
Other	876 (27)	15	735.4	2.0 (1.2–3.4)	463 (33)	240.9
Sex worker	1948 (60)	62	2384.8	2.6 (2.0–3.3)	831 (60)	417.1
Saloon/bar/lodge	342 (11)	25	364.9	6.9 (4.6–10.1)	75 (5)	36.9
Fisher folk	89 (3)	4	153.0	2.6 (1.0–7.0)	17 (1)	8.7
**Education level**						
≤Primary	1538 (47)	60	1980.1	3.0 (2.4–3.9)	546 (39)	274.5
≥Secondary	1717 (53)	46	1658.0	2.8 (2.1–3.7)	840 (61)	429.1
**Marital status**						
Single	2020 (62)	73	2472.2	3.0 (2.3–3.7)	569 (41)	283.1
In relationship/married/cohabiting	736 (23)	16	656.2	2.4 (1.5–4.0)	548 (40)	284.3
Divorced/separated/widowed	499 (15)	17	509.7	3.3 (2.1–5.4)	269 (19)	136.2
**Baseline Behavioural risk (last 3** **months)**						
**Used a condom at last sex**						
No	2469 (76)	78	2598.4	3.0 (2.4–3.7)	*Not collected*	
Yes	786 (24)	28	1039.7	2.7 (1.9–3.9)		
**Had transactional sex**					*Collected as unprotected transactional sex*	
No	574 (18)	17	521.2	3.3 (2.0–5.2)	340 (25)	179.0
Yes	2681 (82)	89	3116.9	2.9 (2.3–3.5)	1046 (75)	524.6
**Sex after using recreational drugs**						
No	2805 (86)	89	3195.8	2.8 (2.3–3.4)	1215 (88)	615.4
Yes	450 (14)	17	442.3	3.8 (2.4–6.2)	171 (12)	88.2
**Number of partners in the last 3** **months at baseline**						
≤5	1204 (37)	40	1190.2	3.4 (2.5–4.6)	525 (38)	273.4
≥6	2051 (63)	66	2447.9	2.7 (2.1–3.4)	861 (62)	430.2
**Diagnosed/treated for an STI in last 3** **months at baseline**						
No	2676 (82)	89	3148.2	2.8 (2.3–3.5)	1029 (74)	520.5
Yes	579 (18)	17	489.9	3.5 (2.2–5.6)	357 (26)	183.1

^a^PY: Person years; IR: Incidence rate.

b Participants in the category ‘0 (N/A)’ of ‘Time between cohort enrolment and trial enrolment (months)’ were those who were directly enrolled in the trial and did not have follow-up visits in the registration cohort study.

c A participant could be counted in more than one row for the variables time in follow-up (years) in the PrEPVacc study, and calendar period. For example, they could contribute person years to both the first and second years of follow-up, or 2018 and 2019, hence the total percentages for each of these variables add up to more than 100%. Trial participants who had previously been registration cohort participants, were considered to have been enrolled in the PrEPVacc study on their dates of enrolment in the registration cohort, i.e., considered to be further along in follow-up.

d Data from the Durban Phoenix site for the registration cohort was included in the analyses, despite only the Durban Verulam site contributing data to the trial.

### Predictors of HIV incidence in the registration cohort

More than 3638 PYFU, 106 participants were diagnosed with HIV, an overall incidence rate of 2.9/100 PYFU (95% CI: 2.4–3.5) (Table 1). There was variation in HIV incidence by study site (p = 0.058): 2.2/100 PYFU in Dar es Salaam, 2.7/100 PYFU in Masaka, 3.2/100 PYFU in Durban, 4.6/100 PYFU in Mbeya. Female participants were at much higher risk of acquiring HIV (adjusted rate ratio (aRR) = 5.04, p = 0.004), and there was marginal evidence that participants older than 24 years were at lower risk (aRR = 0.69, p = 0.105).

**Figure 2.**
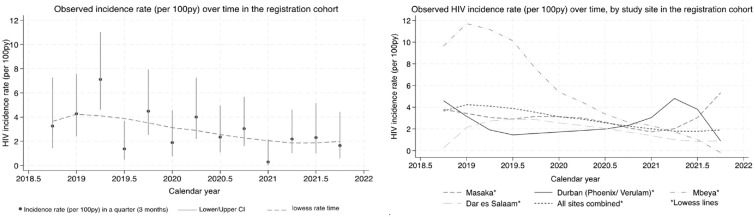
Incidence rate by calendar month in the registration cohort. *The first and last calendar year categories are ≤2018.75 and ≥2021.75 respectively in both graphs.

HIV incidence declined steeply over calendar time, from 4.3/100 PFYU in 2019 to 1.3/100 PYFU in 2021 and 0.4/100 PYFU in 2022/23 (Figure 2). A spike in the number of cases was observed in the first half of 2019, mainly concentrated in the Mbeya site (Figure 2). This corresponds to an overall 37% (95% CI: 19–51) decline per year (Supplementary Table S1). Further analysis indicated that this effect appeared to vary by study site (interaction p = 0.061), with a more pronounced decline in the East African sites than in the South African (Table 2, Supplementary Table S1). Models allowing for non-linear effects (on the log scale) of calendar time were also investigated but showed no material improvement in fit to the data (not shown).

**Table 2.** Associations of socio-demographic variables, behavioural risk factors and time-related predictors (calendar period, time in follow-up) with HIV incidence in the registration cohort.

Characteristic	Univariable analysis		Multi-variable analysis (Option 2)^a^	
	IRR (95% CI)	P value	aIRR (95% CI)	P value
**Site** ^b^		0.020		0.058
Dar es Salaam, Tanzania	Ref		Ref	
Masaka, Uganda	1.23 (0.73–2.06)		1.22 (0.33–4.55)	
Phoenix/ Verulam, South Africa	1.48 (0.76–2.85)		2.14 (0.38–12.14)	
Mbeya, Tanzania	2.11 (1.32–3.37)		3.89 (1.33–11.32)	
**Site specific calendar period effect (1** **year increase)**				0.061^c^
Dar es Salaam, Tanzania	0.67 (0.45–1.01)	0.054	0.58 (0.34–0.96)	
Masaka, Uganda	0.82 (0.51–1.31)	0.406	0.75 (0.45–1.25)	
Phoenix/Verulam, South Africa	1.00 (0.65–1.52)	0.988	0.87 (0.57–1.32)	
Mbeya, Tanzania	0.42 (0.28–0.63)	p < 0.001	0.39 (0.25–0.62)	
**Time in follow-up**		0.023		0.013
0.00–1.00 years	Ref		Ref	
1.01–2.00 years	1.25 (0.83–1.88)		1.91 (1.19–3.07)	
2.01 or higher	0.40 (0.16–1.00)		1.02 (0.35–2.97)	
**Gender**		0.006		0.004
Male	Ref		Ref	
Female	3.16 (1.39–7.2)		5.04 (1.71–14.82)	
**Age**		0.042		0.105
≤24 years	Ref		Ref	
>24 years	0.67 (0.45–0.99)		0.69 (0.46–1.03)	
**Occupation**		p < 0.001		0.104
Other	Ref		Ref	
Sex worker	1.27 (0.73–2.24)		1.61 (0.48–5.38)	
Saloon/bar/lodge	3.36 (1.77–6.37)		2.65 (0.83–8.44)	
Fisher folk	1.28 (0.43–3.86)		3.60 (0.85–15.24)	
**Sex after using recreational drugs**		0.224		0.028
No	Ref		Ref	
Yes	1.38 (0.82–2.32)		1.83 (1.07–3.12)	
**PY accrued during “COVID” (27 March 2020–30 July 2021)**		0.047	Ref 1.09 (0.62–1.90)	0.765
No	Ref			
Yes	0.67 (0.45–0.99)			

PY: person-years; IRR: incidence rate ratio; aIRR: adjusted incidence rate ratio. Multi-variable analyses adjusted for site, calendar period, time in follow-up, gender, age, occupation, sex after using recreational drugs.

a Alternative options for multi-variable analysis models have been provided in supplementary analyses.

b At multi-variable analysis, the site effects (aIRRs) presented are those during the first calendar year of follow-up, that is, July 2018 to June 2019.

c The p value presented is from the likelihood ratio test comparing a model with a study site calendar period interaction term and a model without the interaction term.

Because of the strong effect of calendar time, the multi-variable analysis which adjusts for this effect was considered more reliable for assessing the effect of other predictors. In particular, an inverted U-shaped pattern was observed for the time-dependent predictor, duration in follow-up, with an approximately two-fold higher incidence for the period 1–2 years compared with less than 1 year or more than 2 years. In univariable analysis, the period of the COVID-19 epidemic was associated with a 33% (95% CI: 1–55) lower HIV incidence, but this effect was lost in adjusted analyses, likely due to the strong correlation with the calendar period effect.

### Counterfactual predicted HIV incidence in the trial population

In view of the very strong calendar time effect, and uncertainty regarding the best way to model this, a wide range of sensitivity analyses were performed for predicting the counterfactual HIV incidence in the trial population (Table 3). Other variables included were study site, time in follow-up, age group, gender, occupation, and sex after using recreational drugs.

**Table 3.** Sensitivity analyses: varying estimates of HIV incidence under different assumptions about the association between HIV incidence and calendar period.

Alternative assumptions	Expected incidence rate (per 100 PY) in the PrEP trial by site; expected cases				Expected incidence rate/100 PY (95% CI) in the PrEP trial^a^; expected cases.	Comment
	Dar es Salaam	Masaka	Verulam	Mbeya	Overall	
1) Assuming a null calendar period effect	2.1; 1.9	2.6; 5.5	3.8; 7.1	3.5; 7.4	3.1 (2.1–4.4); 21.8	The predicted placebo HIV incidence rates were highly sensitive to changes in the assumptions made about the calendar period effect.
2) Calendar period effect assumed site-specific	0.7; 0.6	1.4; 3.0	3.4; 6.4	0.5; 1.1	1.6 (0.6–4.2); 11.3	
3) Calendar period effect assumed region specific	0.5; 0.5	0.8; 1.7	3.3; 6.2	0.9; 1.9	1.5 (0.5- 3.1); 10.6	
4) Calendar period effect assumed uniform across sites	0.8; 0.7	1.1; 2.3	1.6; 3.0	1.3; 2.8	1.2 (0.4–2.6); 8.4	
5a) Assuming a null calendar period effect AND variable representing effect of COVID pandemic included	2.5; 2.3	3.0; 6.3	4.3; 8.0	4.4; 9.4	3.7 (2.4–5.5); 26.0	
5b) Calendar period effect assumed site-specific AND variable representing effect of COVID pandemic included	0.6; 0.6	1.4; 3.0	3.2; 6.0	0.4; 0.9	1.5 (0.4-4.1); 10.6	
6a) Data restricted to the more recent period (2020 onwards) and calendar period effect assumed site-specific	1.4; 1.3	3.5; 7.4	1.6; 3.0	0.9; 1.9	1.9 (0.6–7.9); 13.4	
6b) Data restricted to the more recent period (2020 onwards) and assuming a null calendar period effect	1.3; 1.2	1.7; 3.6	5.3; 9.9	1.6; 3.4	2.6 (1.3–4.6); 18.3	

(Total PY observed in the PrEP trial: 703.6 PY Dar es Salaam 92.6 PY; Masaka 211.1 PY; Verulam 187.2 PY; Mbeya 212.6 PY).

a CIs were estimated using bootstrapping methods, resampling participants with replacement from the registration cohort and repeatedly re-estimating the parameters of the prediction model.

The model with a null calendar period effect predicted an incidence rate of 3.1/100 PYFU (95% CI: 2.1–4.4) in a counterfactual trial placebo group (Table 3). This model provides a benchmark and shows the outcome if the effect of calendar period had not been included. The model which assumed a uniform calendar period effect across sites predicted an incidence rate of 1.2/100 PYFU (95% CI: 0.4–2.6). A slightly higher rate was observed for the models with site-specific calendar period effects (1.6/100 PYFU, 95% CI: 0.6–4.2) or region-specific calendar period effects (1.5/100 PYFU, 95% CI: 0.5–3.1). For the analyses which used only the more recent registration cohort data (2020 onwards) an estimate of 2.6/100 PYFU (95% CI: 1.3–4.6) was reached when assuming a null calendar period effect and 1.9/100 PYFU (95% CI: 0.6–7.9) when assuming site-specific calendar period effects. We also conducted additional analyses considering the possibility that the sharp spike in 2018–2019 (Figure 2) observed at the Mbeya site was atypical, and that the subsequent sharp drop in incidence at that site should not inform the calendar effect. We assumed that the site had a calendar effect similar to that at the other sites. (Results are presented in Supplementary Table 2).

As expected, given its weak effect in the multi-variable analysis, incorporating a binary COVID-19 epidemic effect in the model made little difference to the predicted counterfactual HIV incidence. Finally, PrEP uptake in the cohort was low (8.1% of participants reported ever using it) and was not significantly associated with HIV incidence (incidence rate ratio = 0.56 (95% CI: 0.27–1.15)). Sensitivity analyses excluding participants who ever started PrEP did not substantially influence estimates of predicted counterfactual placebo incidence (Supplementary Table 3).

### Lessons learned from application of the registration cohort approach

We learnt several lessons during the conduct and analysis of the registration cohort for the PrEPVacc trial. First, estimation of the counterfactual placebo incidence rate for the trial using the cohort data was challenging because of the considerable uncertainty around HIV incidence calendar trends and the lack of contemporaneous data to the trial. Future studies using the registration cohort approach should aim to ensure close temporal overlap of the cohort and the trial as far as possible to minimise calendar period differences.

Second, if intending to control for calendar trends to inform predictions, substantial person-years need to be accrued to allow robust analyses of the effects of calendar period, time in follow-up and other variables. Of note, COVID-19 disruptions may have influenced HIV incidence in our study population and subsequently the observed calendar trends. If these disruptions had not occurred, it is possible that the registration cohort approach would have been simpler to use with limited adjustments needed for the calendar effect.

Third, data for potential predictors should be collected in as similar a format as possible in the registration cohort and the trial to facilitate combination of the data sets. This would minimise missing data or ‘misclassification’ of data which can occur when combining data fields that are not identical.

In addition, strategies for validation or confirmation of the estimated counterfactual placebo incidence estimates from the registration cohort approach with estimates from other estimation approaches need to be planned early on to address the uncertainty that may arise because of prediction model choices.

Finally, screening procedures and eligibility criteria for the registration cohort could be made to mimic those of the trial to ensure that the two populations are as comparable as possible. However, this can be challenging to implement, as the registration cohort may be set up to also achieve additional objectives other than simply estimating a counterfactual placebo incidence rate. For the PrEPVacc registration cohort, one of its objectives was to progressively shift towards groups at even higher risk of HIV in preparation for the trial. This included early termination of individuals considered to be at lower risk, closing a site likely to have a population at lower risk among others, which resulted in demographic and risk behaviour differences in the cohort and trial populations.

External controls have been used in clinical trials outside HIV prevention for decades.^18^ Substantial literature is available on their use and should be leveraged^18–23^ when setting up a registration cohort or planning to use an external control.

## Discussion

In principle, the use of a registration cohort should be one of the most straightforward and reliable methods for obtaining an estimate of the counterfactual placebo HIV incidence in a trial. However, the unanticipated large decrease in HIV incidence that we observed over calendar time complicates interpretation in this specific application. Our sensitivity analyses revealed the uncertainty that this induces, with counterfactual placebo incidence rates ranging from 1.2/100 PYFU to 3.7/100 PYFU depending on assumptions. The challenge is to identify which of the assumptions are the most reasonable.

According to the Joint United Nations Programme on HIV/AIDS (UNAIDS), regional HIV incidence data show a much smaller downwards trend over the same period, estimated at ∼10% per calendar year in the general population.^24^ This compares with the overall decline of 37% per calendar year in the registration cohort. There are several possible explanations for this discrepancy: initial enrolment of populations at particularly high risk of HIV infection, risk reduction behavioural counselling provided to study participants, selective loss to follow-up of individuals at higher risk,^25^ or a frailty effect due to the loss from the cohort of the sero-converters who were at higher HIV risk potentially leaving behind a group at lower risk. In our study, lower incidence was observed beyond 2 years of follow-up. Time in follow-up was strongly correlated with calendar period and due to their collinearity yet limited person-years of observation, it was not possible to clearly distinguish their independent effects. Hence, the estimated steep decline in incidence attributed to calendar period should be interpreted cautiously.

The analyses presented in this article have several limitations. First, the COVID-19 epidemic delayed the start of the trial, increasing the time difference between the registration cohort and the trial, and making the early follow-up in the cohort less relevant to our aim. For this reason, there is a strong argument for excluding the follow-up in 2018–19.

Second, because of the delayed start of trial yet limited timeline, cohort participants were recruited into the trial as soon as they became eligible (completed minimum cohort follow-up), also subsequently direct recruitment into the trial was also allowed. This, alongside burdensome trial procedures, resulted in cohort follow-up in most sites essentially ending when randomisation to the trial started. Thus, there was little overlap between the registration cohort and the trial in calendar time, and so we were effectively making extrapolations beyond the observed data of up to 3 years. As non-linear models fitted no better than linear models (on the log scale), we used linear models which therefore assumed that the substantial year-on-year decline in HIV incidence continued throughout trial follow-up. This assumption will be testable at the end of the trial by considering information from the period after the third vaccination, when study PrEP is no longer provided.

Third, only 24% of participants followed up in the registration cohort were subsequently enrolled in the trial. There were no notable differences between the cohort participants’ self-reported risk in comparison to the trial participants, and our models controlled for differences in the baseline demographic and behavioural risk data we collected. However, it is possible that those enrolled in the trial were intrinsically at lower or higher risk of HIV infection, linked to unobserved variables. For example, more settled (less mobile) individuals may have been better able to commit to taking part in the trial. In addition, unlike the registration cohort, the PrEP trial participants had to agree to receive both of the trial interventions, that is, administration of vaccines and an offer of oral PrEP. These interventions could be more attractive to individuals at higher risk of HIV infection.

Fourth, 43% of trial participants were directly enrolled in the trial without any follow-up in the registration cohort, again with the possibility of selection bias. Of note, as this group had zero follow-up in the registration cohort, we assumed that the effect of time in follow-up at enrolment in these participants was equivalent to the estimable 0–1 year category in the registration cohort.

Finally, to adjust for PrEP uptake in the registration cohort, we conducted sensitivity analyses where the participants who took up PrEP (8.1%) were excluded from the counterfactual incidence estimation analyses. There were no substantial differences with estimates from the analyses where they were included. If PrEP uptake and adherence in the cohort were higher, the counterfactual estimation process would have been complicated further as excluding those who took it up could potentially introduce selection bias.

Because of these analytical and interpretational challenges, the PrEPVacc Trial Steering Committee has recommended that the final counterfactual placebo HIV incidence estimation should also include information from the period during the trial when study PrEP is no longer provided. However, this analysis is also not straightforward and will need to consider the potential use of locally provided PrEP outside of the study and whether the vaccines provide any protection against infection. As a follow-up to this article, we intend to publish a manuscript synthesising various counterfactual placebo HIV incidence estimates for the PrEPVacc trial such as using the adherence–efficacy relationship of Truvada and using the PrEPVacc trial data. We will evaluate the implications of the various estimates for the AIR.

Despite these difficulties, we would advocate the use of a registration cohort in future studies, where feasible, since rational interpretation of active-control prevention trials is challenging without some information on the counterfactual placebo incidence. Other suggested approaches, such as using recency assays on baseline sero-prevalent cases, inferring the effectiveness of the control regimen from adherence data, and exploiting ecological associations with the incidence of other sexually transmitted infections data, also have a role but depend heavily on untestable assumptions.

## Supplemental Material

sj-pdf-1-ctj-10.1177_17407745241304721 – Supplemental material for Challenges in estimating the counterfactual placebo HIV incidence rate from a registration cohort: id="math59-00375497 Supplemental material, sj-pdf-1-ctj-10.1177_17407745241304721 for Challenges in estimating the counterfactual placebo HIV incidence rate from a registration cohort: id="math59-00375497 by Sheila Kansiime, Christian Holm Hansen, Eugene Ruzagira, Sheena McCormack, Richard Hayes and David Dunn in Clinical Trials
