# Supplementary material for: Challenges in estimating the counterfactual placebo HIV incidence rate from a registration cohort: The PrEPVacc trial
Source: Clin Trials. 2024 Dec 31;22(3):289–300. doi: 10.1177/17407745241304721 (PMC12092936; doi:10.1177/17407745241304721)
Supplement: sj-pdf-1-ctj-10.1177_17407745241304721 – Supplemental material for Challenges in estimating the counterfactual placebo HIV incidence rate from a registration cohort: id="math59-00375497 [file sj-pdf-1-ctj-10.1177_17407745241304721.pdf]

Supplementary Table 1. Alternative calendar period effects multivariable models

Characteristic	Multivariable analysis (Option 1)		Multivariable analysis (Option 3)		Multivariable analysis (Option 4)		Multivariable analysis (Option 5)	
	aIRR (95% CI)	P-value	aIRR (95% CI)	P-value	aIRR (95% CI)	P-value	aIRR (95% CI)	P-value
**Site**		0.263		0.047		0.038		0.158
Dar es Salaam, Tanzania	Ref		Ref		Ref		Ref	
Masaka, Uganda	1.33 (0.68 – 2.62)		1.87 (0.93 – 3.74)		1.73 (0.87 – 3.45)		1.47 (0.74 – 2.91)	
Phoenix/ Verulam, South Africa	2.39 (0.64 – 8.89)		1.93 (0.38 – 9.85)		4.05 (1.05 – 15.63)		2.46 (0.66 – 9.25)	
Mbeya, Tanzania	1.59 (0.94 – 2.69)		2.16 (1.26 – 3.70)		2.01 (1.18 – 3.45)		1.78 (1.04 – 3.03)	
**Region specific calendar period effect (1 year increase)**				0.065*				
East African sites			0.53 (0.39 – 0.73)					
South African sites	N/A	N/A	0.87 (0.57 – 1.32)		N/A	N/A	N/A	N/A
**Calendar period effect assumed uniform across sites**						P<0.001		
(1 year increase)	N/A	N/A	N/A	N/A	0.63 (0.49 – 0.81)		N/A	N/A
**Time in follow up**		0.048		0.018		0.029		0.046
0.00-1.00 years	Ref		Ref		Ref		Ref	
1.01- 2.00 years	1.34 (0.88 – 2.05)		1.90 (1.18 – 3.04)		1.74 (1.10 – 2.73)		1.51 (0.98 – 2.33)	
2.01 or higher	0.44 (0.18 – 1.11)		1.03 (0.36 – 2.89)		0.81 (0.30 – 2.18)		0.57 (0.22 – 1.49)	
**Gender**		0.004		0.004		0.002		0.004
Male	Ref		Ref		Ref		Ref	
Female	4.94 (1.69 – 14.44)		4.99 (1.70 – 14.70)		5.38 (1.86 – 15.54)		4.86 (1.66 – 14.26)	
**Age**				0.076		0.097		0.090
≤24 years	Ref		Ref		Ref		Ref	
>24 years	0.72 (0.48 – 1.07)	0.101	0.70 (0.47 – 1.04)		0.72 (0.48 – 1.06)		0.71 (0.48 – 1.06)	
**Occupation**		0.007		0.092		0.053		0.016
Other	Ref		Ref		Ref		Ref	
Sex worker	1.27 (0.39 – 4.14)		1.63 (0.49 – 5.45)		1.52 (0.46 – 5.06)		1.35 (0.41 – 4.45)	
Saloon/ bar/ lodge	2.87 (0.93 – 8.92)		2.77 (0.88 – 8.74)		2.78 (0.88 – 8.83)		2.83 (0.90 – 8.91)	
Fisher folk	4.06 (0.97 – 16.90)		3.35 (0.79 – 14.17)		3.66 (0.86 – 15.63)		3.81 (0.90 – 16.03)	
**Sex after using recreational drugs**		0.039		0.028		0.022		0.037
No	Ref		Ref		Ref		Ref	
Yes	1.75 (1.03 – 2.98)		1.82 (1.07 – 3.11)		1.87 (1.10 – 3.18)		1.76 (1.03 – 3.01)	
**PY accrued during "COVID" (27th March 2020-30th July 2021)**				0.781**		0.737**		0.043
No			Ref		Ref		Ref	
Yes	N/A	N/A	1.08 (0.62 – 1.90)		0.92 (0.55 – 1.53)		0.64 (0.42 – 0.99)	

aIRR- Adjusted incidence rate ratio. Multivariable analyses were adjusted for site, time in follow up, gender, age, occupation, sex after using recreational drugs. PY- Person years
Multivariable analysis options (AIC – BIC):
1: Assuming no/ a null calendar period effect calendar period effect (1065.3-1170.9)
2: Calendar period effect assumed site-specific (1051.57 – 1192.35). *(- Multivariable model presented in the main manuscript tables).*
3: Calendar period effect assumed region specific (1051.53 – 1174.71).
4: Calendar period effect assumed uniform across sites (1052.93 – 1167.31).
5: Assuming no/ a null calendar period effect, but with a COVID effect (1063.09 - 1177.48).
*The P-value presented is from the likelihood ratio test comparing a model with a region (EA Vs SA) calendar period interaction term, and a model without the interaction term.
**Variable not included in the final multivariable analysis model.

Supplementary Table 2. Additional sensitivity analysis on calendar trends

The sensitivity analyses presented below were added to consider the possibility that the spike in 2018-2019 (figure 2) observed at the Mbeya site was atypical, and that the subsequent sharp drop at that site should not inform the calendar effect.

**Assumptions**	**Expected incidence rate (per 100PY) in the PrEP trial by site; expected cases**				**Expected incidence rate/ 100py (95% CI) in the PrEP trial**; expected cases.**	**Comment/ limitation**
	**Dar es Salaam**	**Masaka**	**Verulam**	**Mbeya**	**Overall**	
S1) Excluding the Mbeya data completely from the analyses because of its odd calendar trend and assuming a site-specific calendar trend.	0.9; 0.9	1.7;3.2	3.2; 6.0	N/A	-	An overall estimate can't be obtained since the Mbeya site contributed data to the trial and an estimate of the site's incidence isn't available.
S2) Mbeya included, calendar period effect assumed site-specific, with the Mbeya calendar effect replaced in the prediction model with an average from the other sites.	0.7; 0.6	1.4; 3.0	3.4; 6.4	4.7; 10	2.8 (0.8 – 13.8); 20	In the multi-variable model, since the variables are adjusted for one another, the calendar effect at any site influences the incidence rate ratios of the other variables in the model and subsequently predictions at the other sites, hence by only replacing the estimated Mbeya calendar effect at prediction, the calendar trend at the site has not been fully ignored hence the need for S3 below.
S3) Mbeya included, calendar period effect assumed site-specific with the Mbeya trend nullified at generating the multivariable prediction model and also replaced with an average from the other sites at prediction.	0.8; 0.8	1.7; 3.6	3.2; 5.9	1.2; 2.6	1.8 (0.6 – 5.4); 13	Nullifying the Mbeya trend at model building may imply that the estimated incidence rate ratios for other variables in the model are left with uncontrolled confounding from calendar period.

Supplementary Table 3. Sensitivity analyses considering exclusion of participants who ever started PrEP in the registration cohort

**Alternative assumptions on calendar period effect**	**Expected incidence rate (per 100py) in the PrEP trial**; expected cases. (Includes all participants at trial sites in the registration cohort)**	**Expected incidence rate (per 100PY) in the PrEP trial**; expected cases. (Sensitivity analyses excluding participants who ever started PrEP in the registration cohort)**	**Comment**
1) Assuming a null calendar period effect	3.1 (2.1 – 4.4); 21.8	3.4 (2.3 – 4.9); 23.9	Slightly higher incidence predicted
2) Calendar period effect assumed site-specific	1.6 (0.6 – 4.2); 11.3	1.6 (0.6 – 4.6); 11.3	No substantial difference
3) Calendar period effect assumed region specific	1.5 (0.5- 3.1); 10.6	1.5 (0.5 – 3.5); 10.6	No substantial difference
4) Calendar period effect assumed uniform across sites	1.2 (0.4 – 2.6); 8.4	1.2 (0.4 – 2.8); 8.4	No substantial difference

- *(Total py observed in the PrEP trial: 703.6py Dar es Salaam 92.6py; Masaka 211.1py; Verulam 187.2py; Mbeya 212.6py). **CIs were estimated using bootstrapping methods, resampling participants with replacement from the registration cohort and repeatedly re-estimating the parameters of the prediction model.*
